# Geographic distribution of non-clinical *Theileria parva* infection among indigenous cattle populations in contrasting agro-ecological zones of Uganda: implications for control strategies

**DOI:** 10.1186/1756-3305-7-414

**Published:** 2014-09-01

**Authors:** Fredrick Kabi, Charles Masembe, Vincent Muwanika, Halid Kirunda, Riccardo Negrini

**Affiliations:** Department of Environmental Management, Molecular Genetics Laboratory, College of Agricultural and Environmental Sciences, Makerere University, P.O. Box 7062/7298, Kampala, Uganda; National Livestock Resources Research Institute (NaLIRRI), P.O. Box 96, Tororo, Uganda; Department of Biological Sciences, College of Natural Sciences, Makerere University, Box 7062, Kampala, Uganda; Istituto di Zootecnica, Università Cattolica del Sacro Cuore, Institute of Zootechnics, via Emilia Parmense 84, 29122 Piacenza, Italy

**Keywords:** Agro-ecological zones, East Coast Fever, Endemic stability, Non-clinical *T. parva* AEZ gradient, Indigenous cattle, Uganda

## Abstract

**Background:**

Non-clinical *Theileria parva* infection among indigenous cattle occurs upon recovery from primary disease during the first year of life. Continuous exposure to infection through contaminated tick infestations with absence of clinical disease gives rise to endemic stability. Endemic stable populations may become sources of infection if contaminated tick vectors are shared with susceptible exotic cattle. This study aimed at establishing a nationwide distribution of non-clinical *T. parva* infection among indigenous cattle populations to inform novel control strategies.

**Methods:**

The occurrence of non-clinical *T. parva* infection among apparently healthy 925 indigenous cattle from 209 herds spread out in 10 agro-ecological zones (AEZs) was determined using a nested PCR assay. The influence of AEZ, breed, sex, age and farmers’ ranking of ECF importance were interrogated for influence of non-clinical parasite occurrence.

**Results:**

The overall prevalence of non-clinical *T. parva* infection was 30% (278/925). A gradual increase of non-clinical *T. parva* infection was observed ranging from 17% (95% CI: 0.03 – 0.23) to 43% (95% CI: 0.3 – 0.55) in the North Eastern Savannah Grasslands (NESG) to the Western Highland Ranges (WHR) respectively. A similarly associated 18% (95% CI: 0.07 – 0.28) and 35% (95% CI: 0.3 – 0.39) non-clinical parasite prevalence was observed among the East African shorthorn Zebu (EASZ) and Ankole cattle respectively. Average herd level non-clinical *T. parva* prevalence was 28%, ranging from zero to 100%. The likelihood of non-clinical *T. parva* infection was 35.5% greater in the western highlands compared to the northeastern semi-arid AEZs.

**Conclusions:**

Non-clinical *T. parva* occurs countrywide, structured along patterns of AEZ and breed gradients. These findings may guide policy formulation, deployment of integrated control strategies and local cattle improvement programs.

## Background

East coast fever (ECF) is caused by *T. parva* and often exhibits a non-clinical infection among indigenous cattle which have recovered from the primary disease and are continuously exposed to infection, a phenomenon called endemic stability. However exotic dairy cattle genotypes may suffer 100% mortality [1]. Endemic stability is a state of interaction between the host, tick vector and pathogen, whereby calves are infested with contaminated ticks, exhibit non-acute or mild clinical disease, which further develops into a high level of immunity in adult cattle with absence of clinical disease as described by Jonsson et al. [2]. In Uganda, indigenous cattle breeds comprise about 93.3% of the national cattle herd and include the Ankole, East African shorthorn zebu (EASZ), Nganda and their crosses [3]. Their ability to survive under continuous ECF challenge is known to have developed as a result of host parasite evolutionary co-existence dating back to cattle introduction in the disease endemic region [4, 5]. Indigenous cattle populations and contaminated ticks can often be a source of infection to naïve exotic cattle in tick-borne diseases (TBDs) endemic areas [5, 6]. More so where indigenous and exotic cattle are managed in close proximity, resulting into sharing of contaminated ticks, risks of disease transmission and high mortalities [5] among the later.

Urbanisation and improved incomes have stirred high demand for animal proteins in Sub-Saharan Africa (SSA) which mandates keeping livestock of high productivity [7, 8] accompanied with robust disease control approaches. Stakeholders in Uganda’s dairy sector have responded by promoting high yielding exotic cattle breeds [2, 9]. Unfortunately, resource limited dairy cattle farmers do not fully benefit from this innovation due to high disease control expenses and mortality caused by ECF often transmitted from endemic stable indigenous cattle by contaminated ticks [10, 11]. *Theileria parva* is an intracellular protozoan parasite, transmitted by the three-host ixodid tick *Rhipicephalus appendiculatus* (brown ear tick) that is widely spread in 12 countries of SSA*,* occurring where the vector survives [5, 10–13]. The parasite is spread both cyclopropagatively and transstadially by the brown ear ticks which have acquired infection by feeding on infected cattle during the earlier stage of their life cycle [5]. East Coast fever causes high morbidity and mortality particularly among exotic and crossbred cattle which are preferred for improvement of dairy production in SSA [1].

Suitable environmental conditions for *R. appendiculatus*, inappropriate control strategies and abundant substitute tick hosts have favoured the widespread occurrence of ECF [13–15] in SSA. The existence of endemic stability among Ugandan indigenous cattle populations is facilitated by high rates of *T. parva* infection and sero-conversion with rare occurrence of clinical disease [16, 17]. Tick control by acaricide application formerly introduced in the region by colonial governments is the predominant approach for controlling TTBDs [5, 18, 19]. This approach is now liberalised along with other veterinary services [18–20]. Present acaricide use on indigenous cattle [18, 19] aims to reduce primary ECF stress and improve growth vigour to attain early market weight. However, intensive acaricide use among indigenous cattle systems interferes with endemic stability, increasing the risk of occurrence of clinical ECF [5], tick resistance and environmental contamination [5, 19, 21, 22]. The popularity of indigenous cattle and their tolerance to TTBDs in Uganda have been reported [23–28] providing support for conservation and sustainable production. Currently, the control of livestock diseases through vaccine application is being promoted by the Global Alliance for Livestock Veterinary Medicines (GALVmed) [5, 8]. The present ECF vaccine involves an infection and treatment method (ITM) [29–33] known to impart immunity to successive homologous parasite infections. Development of novel control strategies for TTBDs is necessitated by the fact that indigenous cattle are popular and carry abundant ticks [6]. This makes them potential sources of infection, and yet there is urgent need to increase productivity by keeping exotic dairy cattle more so in the advent of veterinary services liberalisation in Uganda.

Prospectus sustainable ECF control should be tailored to the different AEZs and breeds with reduced acaricide use [5, 34–38] and improvement of indigenous cattle. Nationwide non-clinical *T. parva* prevalence and distribution among indigenous cattle populations will provide critical information for policy formulation and ECF control. In this study, a nested PCR assay with high sensitivity and specificity for detection and monitoring of non-clinical *T. parva* infection in cattle [39] was used to determine a countrywide non-clinical parasite distribution among the different cattle populations.

## Methods

### Study area

With a total size of approximately 241,550.7 square kilometres (sq.km), (open water and land: − 41,743.2 199,807.4 sq. km respectively), Uganda lies across the equator in Eastern Africa between longitudes 29 ½° East and 35° East and between latitudes 4 ½° North and ½° South, at an average altitude of 1,100 meters above sea level. The ecological diversity of Uganda is characterised by a wide range of altitudes above sea level, ranging from 620 metres (Albert Nile) to 5,111 metres (Mt. Rwenzori peak). Uganda’s water bodies including Lake Victoria (shared between Uganda, Kenya and Tanzania), Lake Albert and Edward (shared with the Democratic Republic of Congo - DRC), Wamala, Bunyonyi, Katwe, Nakivale, Mburo, Kyoga, George and Bisina influence the AEZs climatic attributes. These lakes are drained by rivers including Aswa, Kagera and the Nile [40], which influence climatic conditions and subsequently provide suitable environmental conditions for TTBDs. This study used the 10 AEZs described on the basis of a fairly uniform socio-economic background and ecological conditions, farming systems and practices [41]. A summary of the 10 AEZs is shown in Table 1.

**Table 1:** **Summary description of the 10 AEZs of Uganda**

AEZ	Annual rainfall (mm)	Altitude (m ASL)	Annual temperature (°C)
**NED**	745	351 – 1,524	12 – 33
**NESG**	1,197	975 – 1,524	15 - 33
**NWSG**	1340	351 – 1,341	15 - 25
**PS**	1,259	351 – 1,341	18 – 33
**KP**	1,200 – 1,450	914 – 1,800	15 – 33
**LVC**	1,200 – 1,450	1,000 – 1,800	15 – 30
**WSG**	1,270	621 – 1,585	15 – 30
**PR**	1,270	129 – 1,524	13 – 30
**SWF**	1,120 – 1,223	129 – 1,524	13 – 30
**WHR**	1,400	1,299 – 3,962	8 – 28

AEZ - Agro-Ecological Zone, NESG - North Eastern Savannah Grasslands, NED - North Eastern Drylands, KP - Kyoga Plains, NWSG - North Western Savannah Grasslands, PSG - Para-Savannah Grasslands, WSG - Western Savannah Grasslands, LVC - Lake Victoria Crescent, PR - Pastoral Rangelands, SWF - South Western Farmlands, WHR - Western Highland Ranges, Adopted from [41]).

Indigenous cattle are present in all the AEZs of Uganda, widely preferred by the local cattle keeping communities for adaptation to the different climatic conditions, low quality feed resources and tolerance to endemic disease challenges [3]. According to the 2009 Livestock Census Report, 26.1% of all households in Uganda owned cattle providing a potential for breed improvement and sustainable production.

### Sample and data collection strategy

**Figure 1:**
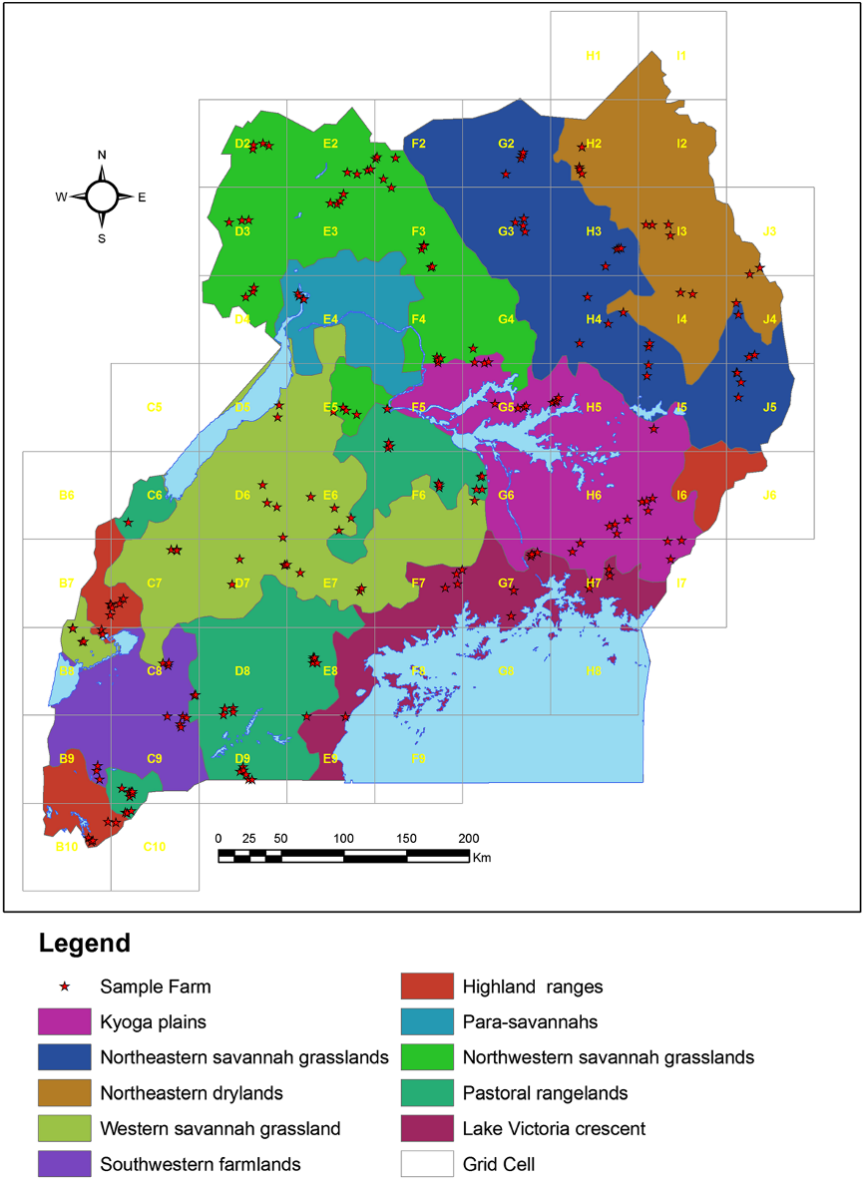
**The distribution of indigenous cattle sample farms (n = 209) in the 10 AEZs of Uganda.** The distribution of the 209 farms/herds where 925 indigenous cattle blood samples were obtained in order to establish the overall distribution of non-clinical *T. parva*. Sample collection was guided by the 50 grid cells (approx. 50 x 50 Km) in which 4–6 farm households were selected. From each household farm/herd 4–5 indigenous cattle were randomly selected. The 10 agro-ecological zones (AEZs) were used to structure the indigenous cattle populations and the non-clinical *T. parva* infection. The sampling strategy was designed by the NextGen Project [43] to enable a landscape data collection.

The samples and data for this study were collected from January 2011 to April 2012, more actively during the rainfall seasons when pastoralists are more available and not moving long distances in search for pasture. The rainfall seasons coincide with high tick burdens and probably high infection of *T. parva*. The sample size (n) was determined using the statistical formula, n = Z^2^ p (1-p)/d^2^, Where n is the sample size, Z is 95% confidence interval (1.96), p is expected prevalence (which was estimated at 50%) and d is margin of error (5%) [42]. Given this formula, a total of 368 cattle was adequate per breed. Given the wide variation in size and cattle population within each AEZ, the sample size per zone was determined to vary from 50 to 150 head of cattle and was guided by grid cells to ensure uniform distribution of sample collection. A total of 925 apparently healthy indigenous cattle were used in this study. The grid cells, location of sampling sites and AEZs are shown in Figure 1. This study embraced a landscape sampling strategy defined by 50 grid cells (approximately 50 × 50 sq. km), enabling an inclusive sampling across the 10 AEZs as designed under the NextGen Project [43] aimed at establishing differences among AEZs. Blood samples were obtained from 925 apparently healthy cattle with no obvious clinical manifestations of ECF comprising 410 Ankole (*B. taurus indicus*) 465 EASZ and 50 Nganda (*B. indicus*). The number of Nganda was quite low due to the small area where they are kept, but were considered since they are EASZ type within central Uganda. An Etrex® global positioning system (GPS) handset device was used for recording the topographical positions of the different sample farms/herds. Locations (latitudes and longitudes) of the 209 farms/ herds were overlaid on the 10 AEZs using ArcGIS® version 10 to show the countrywide pattern of data collection as shown in Figure 1. Short questionnaire interviews were administered to farmers/herd owners to establish the age of sampled cattle (confirmed by dentition), administrative locations, and important diseases recorded in the herd and the main control approaches. The veterinary local extension workers assisted with local language translation and interpretation of the questions to the farmer households. Responses were entered into the record sheets. Before data collection, the questionnaire was tested by veterinary officials among selected cattle farmers and improved by the research team.

### Blood sample collection and total DNA extraction

About 4mls of whole blood was collected by venipuncture from well restrained cattle into well-labelled ethylenediaminetetraacetic acid (EDTA) coated vacutainer tubes and stored on ice in a cool box. Corresponding records of location, ownership, age and sex were entered onto a field data sheet. The iced box with samples was transported to Makerere University Molecular Genetics Laboratory in the department of Environmental Management within 36 hours. At the laboratory, blood was stored at −20°C before DNA extraction. Total DNA was extracted from whole blood using a Qiagen QIAmp® DNA extraction kit (Qiagen-GmbH Hilden Germany) according to the manufactures instructions. The quality of DNA was checked by electrophoresis on 1% agarose gel.

### Nested PCR amplification of *T. parva*

The primer set for detection of *T. parva* based on the p104 antigen gene (Genbank M29954) were obtained from Oligo™ Macrogen Seoul Korea. The sequences of the primary forward and reverse primers 5’-ATT TAA GGA ACC TGA CGT GAC TGC-3’ and 5’-TAA GAT GCC GAC TAT TAA TGA CAC C-3’ respectively. The sequences of the nested forward and reverse primers were 5’-GGC CAA GGT CTC CTT CAG ATT ACG-3’ and 5’-TGG GTG TGT TTC CTC GTC ATC TGC-3’ respectively, Odongo et al. [39]. This primer set was designed to amplify a 277 bp fragment which is a highly conserved segment of p104 gene, a specific and sensitive target for *T. parva* detection. *Theileria parva* muguga strain control DNA was kindly donated by Bio-sciences eastern and central Africa (BecA), ILRI Hub, Nairobi. Primary and nested PCR assays were performed in 20 μl AccuPower® PCR PreMix Bioneer® tubes (USA) as previously described by Odongo et al. [39], with minor modifications. The PCR products were electrophoresed on 1.5% agarose (Bio Tolls Inc. Japan), stained with 5% Ethidium bromide™ (Biotium, Inc., USA) Agarose gel for 30 minutes and the positive samples were visualised as a 277 bp band on the agarose gel under UV trans-illuminator.

### Data analysis

Raw data on *T. parva* prevalence, age group, sex, breed were entered into Microsoft Excel® 2010, exported to Stata® ver. 12 (2012) statistical package, cleaned and coded for computation. Mean prevalence of *T. parva* among the different AEZs breed, sexes, age groups were computed at 95% confidence interval (CI). A multivariate mixed logistic regression model was used to estimate the odds ratio (OR) for the risk of *T. parva* infection with adjustment for AEZ, breed, gender and age group.

### Plotting of the prevalence data on the map of Uganda

**Figure 2:**
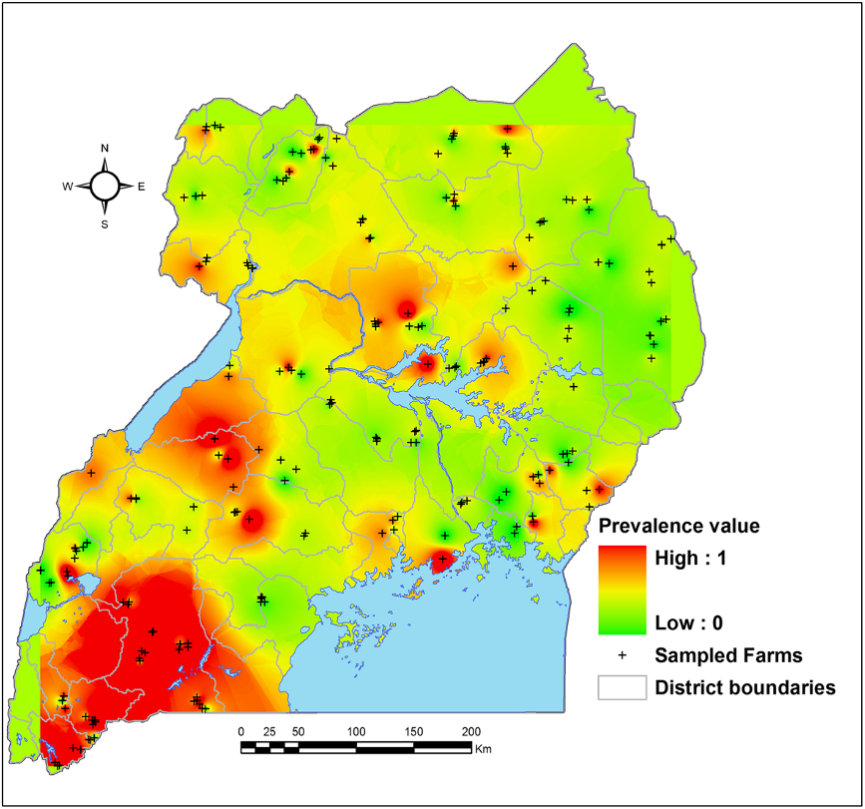
**The spatial distribution of non-clinical** ***T. parva*** **infection among indigenous cattle populations in Uganda: January 2011 to April 2012.** The spatial distribution of non-clinical *T. parva* infection derived from apparently healthy indigenous cattle populations in Uganda (January 2011 to April 2012) was interpolated using 209 study herd prevalence values to create a nation-wide spatial effect. An inverse distance weighted interpolation (IDWI) on the spatial analyst extension of ArcMap 10 was used to generate the continuous non-clinical *T. parva* infection distribution map on a red colour for higher and green for lower parasite occurrence. Parameters were set so that for each pixel in the continuous raster an average prevalence was calculated based on all non-clinical *T. parva* prevalence values at herd level. Being a weighted average, the weights was higher for herds near the pixel (red) and lower for more distant herds (green). An appropriate exponent value of 20 km was chosen to generate a continuous non-clinical *T. parva* prevalence map over the 209 individual herd prevalence values.

Using the Geographic Information System (GIS) records from the field and the ECF infection frequency per herd, the inverse distance weighted interpolation of ArcMap technology was used to create a continuous non-clinical *T. parva* infection spatial distribution map of Uganda in order to display the parasite distribution across the across the entire country (Figure 2).

### Ethical clearance

This study was ethically cleared by Makerere University Institute of Environment and Natural Resources (MUIENR) and approved by the higher degree committee of Makerere University. Permission to undertake the study was obtained from the Uganda National Council for Science and Technology (UNCST) reference number NS 325. The district veterinary personnel and farmers provided oral consent for the use of their cattle and information for the study.

## Results

The present study used data from apparently healthy 925 indigenous cattle (50 Nganda, 410 Ankole and 465 EASZ) from 10 AEZs and 209 herds spread throughout Uganda.

The screening of the blood samples using a nested PCR assay revealed an overall non-clinical *T. parva* infection prevalence of 30% (248/925). The prevalence of non-clinical *T. parva* infection at herd level was highly variable ranging from 0.0% to 100%, however the mean herd prevalence of 28% was observed. The distribution of non-clinical *T. parva* infection across the ten AEZs can be categorised into three: − low (17 – 18), medium (22 – 27) and high (36 – 43) prevalence zones. The low prevalence AEZs were NESG and NED with 17% (95% CI: 0.1 – 0.23) and 18% (95% CI: 0.08 – 0.27) respectively. The medium non-clinical *T. parva* infection prevalence zones were composed of KP, NWSG, PSG and WSG i.e., 22% (95% CI: 0.14 – 0.28), 25% (95% CI: 0.17 – 0.31), 25% (95% CI: 0.03 - 0.46) 26% (95% CI: 0.17 - 0.33) and LVC 27% (0.15 - 0.38) respectively. The high non-clinical *T. parva* infection prevalence zones included PR, SWF and WHR (36% (95% CI: 0.28 – 0.43), 39% (95% CI: 0.22 – 0.55), 43% (95% CI: 0.3 – 0.55) respectively. Table 2 shows the detailed distribution of non-clinical *T. parva* infection across the 10 AEZs.

**Table 2:** **Distribution and risk of non-clinical** ***T. parva*** **infection among the 10 AEZs**

AEZ	Districts sampled	n = 925	Non-clinical ***T. parva***occurrence (95% CI)	Odds ratio (OR)	***p***-value
**NESG**	Pader, Kitgum, Katakwi, Abim	127	17 (0.1 - 0.23)	Reference	
**NED**	Northeastern Kotido, eastern Kitgum, northern Nakapiripiriti	61	18 (0.08 - 0.27)	1.05	1.00
**KP**	Iganga, northern Bugiri, Tororo, Kaberamaido	134	22 (0.14 - 0.28)	1.27	0.53
**NWSG**	Adjumani, western Nebbi, Arua, Yumbe, northern Gulu, northern Apac	155	25 (0.17 - 0.31)	1.55	0.14
**PSG**	Eastern Nebbi, southwestern Gulu, western Masindi	16	25 (0.03 - 0.46)	1.59	0.49
**WSG**	Hoima, Kibaale, Kyenjonjo,	113	26 (0.17 - 0.33)	1.64	0.15
**LVC**	Southern Masaka, Bukomansimbi, Buikwe, Mpigi, Jinja, Mayuge	56	27 (0.15 - 0.38)	1.74	0.16
**PR**	Masindi, Nakasongola, southern Mubende, eastern Mbarara, southern Ntungamo	166	36 (0.28 - 0.43)	2.70	0.0004**
**SWF**	western Mbarara, northern Ntungamo, Rukungiri	36	39 (0.22 - 0.62)	3.03	0.0110**
**WHR**	Kabale, Kasese, western Kyenjonjo	61	43 (0.3 - 0.73)	3.55	0.0003**

AEZ - Agro-Ecological Zone, NESG - North Eastern Savannah Grasslands, NED - North Eastern Drylands, KP - Kyoga Plains, NWSG - North Western Savannah Grasslands, PSG - Para-Savannah Grasslands, WSG - Western Savannah Grasslands, LVC - Lake Victoria Crescent, PR - Pastoral Rangelands, SWF - South Western Farmlands, WHR - Western Highland Ranges, **Significantly difference from the reference (Ref. p < 0.05).

The nation-wide distribution of non-clinical *T. parva* infections among indigenous cattle exhibited a gradual decrease with high prevalence occurring in the highland AEZs, while the low incidences exhibited in the eastern savannah grasslands and semi-arid drylands zone. Figure 2 displays the spatial distribution of non-clinical *T. parva* infection across the whole country.

### The distribution of non-clinical *T. parva*(ECF) infection by age, sex and breed

The distribution of non-clinical *T. parva* infection by age, sex and breed type among the study cattle were computed and is shown in Table 3. The prevalence of non-clinical *T. parva* infection decreased with increase in age thus 36% (7 – 24), 30% (25 – 36), 28% (37 – 72) and 25% (73 – 192). The occurrence of non-clinical *T. parva* infection was comparable among the female and males thus 26% and 27% respectively. The prevalence of non-clinical *T. parva* infection was significantly higher among the Ankole (35%) as compared to the Nganda (18%) and EASZ (21%) cattle, although the Nganda sample size was comparatively low.

**Table 3:** **Results of multivariate regression for the distribution and risk of non-clinical** ***T. parva*** **infection by age, sex and breed**

Study population attributes	n = 925	Number of positives by nested PCR	Prevalence (%) 95% CI	Odds ratio (OR)	***p***-value
**a) Age**					
**7-24**	70	25	36	Ref	
25-36	83	46	30 (0.29 - 1.29)	0.71	0.24
37 - 72	332	94	28 (0.4 - 1.28)	0.61	0.21
73 - 192	440	108	25 (0.33 – 1.04)	0.59	0.05*
**b) Gender**					
Female	801	216	27	Ref	
Male	124	32	26 (0.68 – 1.69)	1.06	0.82
**c) Breed type**					
EASZ	465	96	21	Ref	
Ankole	410	143	35 (1.5 – 2.8)	2.06	0.000**
Nganda	50	9	18 (0.34 – 1.84)	0.84	0.85

EASZ - East African shorthorn Zebu, *significant **Highly significant in relation to reference (Ref. = reference, p ≤ 0.05).

### Farmers’ ranking of the economic importance of ECF in comparison to other diseases

The outcome of questionnaire interviews administered to 209 farmers seeking to establish the history and occurrence of at least three diseases in the previous five years on study farms and rank the order of economic importance of these diseases is shown in Table 4. Eighty percent of all the responses indicated that ECF was the most important disease on their farms. This information was validated by the district veterinary personnel.

**Table 4:** **Farmers’ ranking of the economic importance of ECF and other common diseases in their herds**

AEZ		Farmers interviewed (n = 209)	Disease rank (economic importance)			
			1	2	3	4
1	WHR	16	TBDs	ECF	FMD	NR
2	KP	36	ECF	TBDs	Nagana	Helminths
3	LVC	15	ECF	Nagana	TBDs	Helminths
4	NED	13	ECF	TBDs	Nagana	NR
5	NESG	27	ECF	Nagana	TBDS	Helminths
6	NWSG	36	ECF	TBDs	Nagana	Helminths
7	PSG	4	Nagana	TBDs	Helminths	NR
8	PR	38	ECF	TBDs	FMD	Helminths
9	SWF	9	ECF	FMD	TBD	NR
10	WSG	24	ECF	TBDs	FMD	Helminths

ECF – East Coast Fever, TBDs – Tick-borne diseases, FMD – Foot and Mouth disease, NR – No response, AEZ - Agro-Ecological Zone, NESG - North Eastern Savannah Grasslands, NED - North Eastern Drylands, KP - Kyoga Plains, NWSG - North Western Savannah Grasslands, PSG - Para-Savannah Grasslands, WSG - Western Savannah Grasslands, LVC - Lake Victoria Crescent, PR - Pastoral Rangelands, SWF - South Western Farmlands, WHR - Western Highland Ranges.

## Discussion

The present study determined the geographic distribution of non-clinical *T. parva* infection based on samples collected from January 2011 to April 2012 among traditionally managed cattle. The field visits coincided with the long and short rainfall seasons when pastoral cattle keepers did not have to trek long distances in search of pastures. It is also during these periods that tick burdens are high with possibilities of comparatively high *T. parva* infection challenge [13, 14, 36]. The overall prevalence of non-clinical *T. parva* (30%) infection established in this study is within the expected limits given the fact that indigenous cattle are frequent carriers of piroplasms associated with non-clinical low parasitaemia. Similar nationwide prevalence surveys carried out in neighbouring Rwanda between 1998 and 2003, based on p104 *T. parva* specific gene and 18S assays established comparable prevalence ranges of 25.3% to 27.1% [36]. The authors observed correlations between the p104 *T. parva* specific gene and 18S assays prevalence and no significant differences between the different seasons. The occurrence of ECF is widespread in Uganda and economically important as observed from the farmers’ responses, which favours deployment of strategic control approaches.

Occurrence of non-clinical *T. parva* infection in this study exhibited a wide range of variation among the different AEZs i.e., from a low 17% to high 43%, displaying a significant increase from the NESG and NED to the southwestern (SWR, WHR, PR) AEZs respectively. A similar observation was made at herd level prevalence of non-clinical *T. parva* infection. This could be a reflection of differences in the intensity of *R. appendiculatus* activity, indigenous cattle breed resistance and control practices on individual farms in the different AEZs as recently reported by Muhanguzi et al. [44]. Within the different AEZs, various ecological, weather conditions and farmers’ management systems affect tick multiplication which may vary according to the season of the year. Higher rainfall patterns, suitable environmental temperatures and abundant wildlife coupled with pastoral and open grazing systems practiced in southwestern and western Uganda favours increased cattle – tick activity [45]. Similar conditions are prevalent within the central, southeastern and Kyoga plains, however, farmers here practice higher levels of crop-livestock farming systems limiting extensive cattle movements and exposure to ticks [11, 44]. Under these conditions traditionally managed cattle have a continuous exposure to moderate tick burdens and *T. parva* exposure, a scenario we loosely associate with endemic stability to ECF. Similar situations have been reported in western Kenya and Uganda among smallholder cattle farmers [5, 11].

While the northeastern drylands and northern savannah grassland conditions are comparatively less suitable for tick survival and multiplication. Moreover, cattle keepers practice seasonal movement patterns in search of grazing pastures but in such a way as to avoid conditions leading to higher exposure to ticks especially in the rainfall seasons [46].

Recently, Gachohi and others [5] in a review of ECF infection observed related trends which have been referred to as ECF AEZ gradient in Kenya. Similarly, the existence of tick marginal hot and dry conditions, with sparse grasslands comparable to the NED and NESG zones are responsible for reduced *R. appendiculatus* activity and consequently lowered *T. parva* challenge, resulting into a low non-clinical *T. parva* infection zone. Similar trends have also been observed in area-wide *T. parva* prevalence studies in neighbouring Rwanda [36], where *T. parva* infection was observed to be higher in the high lands and lower in the lowlands. However, higher rainfall as previously experienced under the El-niño conditions in the drier AEZs could result into a drastic upsurge of insects and consequently vector-borne infections as has been reported [37, 38] in this region.

It is worth mentioning here that sharing of pastures with wildlife and prolonged dry season stresses (grazing indigenous cattle) associated with pasture scarcity, risks infection with non-homologous *T. parva* strains from wildlife resulting into acute ECF outbreaks and higher mortalities as was observed by Ocaido et al. [45]. We loosely associate this with breakdown of endemic stability commonly reported during the dry seasons. Within the pastoral rangelands, commercial food production has reduced availability of natural pasture grasslands and denied continuous contact of ticks with indigenous cattle [47] which also interferes with endemic stability and most probably increases the incidence of clinical *T. parva* infection within cattle populations [5].

The current study has observed significantly higher non-clinical *T. parva* infection among the Ankole than the EASZ cattle populations. The occurrence of higher non-clinical *T. parva* infection among the Ankole cattle is most likely associated with increased tick activity resulting from sharing of pastures with wildlife species such as buffaloes and antelopes [45]. Additionally, suitable rainfall conditions create adequate moisture for tick multiplication and survival. The Nganda cattle displayed intermediate non-clinical *T. parva* infection prevalence. This could be due to the AEZs attributes and farmers’ management practices under which these cattle are kept. However, their sample size was comparatively lower in comparison to other cattle breeds in this study. On the other hand, unsuitable tick habitat conditions, routine pastoral migrations associated with rainfall seasons and pasture availability, practiced in the NED and NESG aids to avoid higher tick burdens on cattle [46]. This consequently results into comparatively reduced *T. parva* infection challenges among the EASZ cattle populations. In summary, marginal tick conditions, seasonal cattle movements and indigenous cattle resistance to TTBDs complement each other to maintain a comparatively lower non-clinical *T. parva* occurrence among the EASZ cattle populations.

This study also established a higher non-clinical *T. parva* infection prevalence among the lower age group (though marginal significant) and the oldest group as previously reported [13, 14]. This is a reflection of a lower ability to control *T. parva* infection during primary disease challenges which tends to improve in older cattle.

## Conclusions

This study has revealed the current geographic and indigenous cattle breed patterns of non-clinical *T. parva* infection gradients in Uganda. The gradient of non-clinical *T. parva* infection is influenced by AEZ, breed and farmer cattle management activities. These baselines are critical for development, deployment and monitoring of integrated tick control strategies. These results further motivate indigenous cattle improvement and conservation with *T. parva* infection tolerance.

## Abbreviations

DRC: Democratic Republic of Congo
ECF: East Coast Fever
GALVmed: Global Alliance for Livestock Veterinary Medicines
GPS: Global positioning system
ITM: Infection and treatment method
NaLIRRI: National Livestock Resources Research Institute
SSA: Sub-Saharan Africa
TTBDs: Ticks and Tick-borne diseases.
